# Real-time Concealed Object Detection from Passive Millimeter Wave Images Based on the YOLOv3 Algorithm

**DOI:** 10.3390/s20061678

**Published:** 2020-03-17

**Authors:** Lei Pang, Hui Liu, Yang Chen, Jungang Miao

**Affiliations:** 1 School of Geomatics and Urban Spatial Informatics, Beijing University of Civil Engineering and Architecture, Beijing 100044, China; panglei@bucea.edu.cn (L.P.); chenyang@elensdata.com (Y.C.); 2 School of Electronic and Information Engineering, Beihang University, Beijing 100191, China; jmiaobremen@buaa.edu.cn

**Keywords:** concealed object detection, passive millimeter wave, deep learning, YOLOv3, neural network, real-time

## Abstract

The detection of objects concealed under people’s clothing is a very challenging task, which has crucial applications for security. When testing the human body for metal contraband, the concealed targets are usually small in size and are required to be detected within a few seconds. Focusing on weapon detection, this paper proposes using a real-time detection method for detecting concealed metallic weapons on the human body applied to passive millimeter wave (PMMW) imagery based on the You Only Look Once (YOLO) algorithm, YOLOv3, and a small sample dataset. The experimental results from YOLOv3-13, YOLOv3-53, and Single Shot MultiBox Detector (SSD) algorithm, SSD-VGG16, are compared ultimately, using the same PMMW dataset. For the perspective of detection accuracy, detection speed, and computation resource, it shows that the YOLOv3-53 model had a detection speed of 36 frames per second (FPS) and a mean average precision (mAP) of 95% on a GPU-1080Ti computer, more effective and feasible for the real-time detection of weapon contraband on human body for PMMW images, even with small sample data.

## 1. Introduction

The detection of concealed targets under people’s clothing is essential for public security. With the unique advantage of penetrating most materials, except for metal and water, millimeter wave imaging systems have been employed for concealed target visualization without privacy concerns [1]. Different from active modes, the millimeter sensor usually relies on its strong penetrability, and targets can be identified through their naturally emitted and reflected radiations, detected by using a Passive Millimeter Wave imaging system [2,3,4,5,6]. This leads to less damage to human health and is more suitable for the application of security at airports, subway stations, railway stations, conference places, etc. However, a number of factors, including passive millimeter wave (PMMW) image quality as well as unknown positions, shapes, and sizes of hidden objects, make this task challenging. Numerous image processing techniques, such as image denoising [7], image fusion [8], segmentation [9,10], classification [11,12], object detection and recognition [13,14,15,16] etc., have been developed and applied to PMMW imagery in the last few decades. However, the imaging environment and the imaging hardware limitations usually result in low spatial resolution and contrast for PMMW images. In addition, the information content of a single frame of contraband is less and contains a lot of noise, so it is difficult to obtain reliable and effective results by directly detecting the contraband of each frame of image. The above methods of PMMW data processing are mainly used to perform filtering, threshold segmentation, or edge detection to realize the detection of hidden objects of sequence images. Experiments have shown that they have a certain processing capacity for sequencing PMMW images. Nevertheless, based on single frame processing, most of these above methods have two obvious shortcomings. One is that, with the increase of large passenger flow application scenarios, the data processing speed is too slow to meet the requirements for target recognition and real-time detection [17,18]. The other is that the extraction of object contour is not accurate enough, which is easy to produce ambiguity, resulting in the need for more computation and identification costs.

Due to the size of concealed weapons under people’s clothing usually being very small, it is more difficult to identify them immediately from centimeter-level resolution PMMW images. Especially for some application scenarios and equipment with large passenger flow, a rapid recognition method of some types of concealed weapon targets on human bodies using a small sample dataset, even in no more than 3 seconds, is required to guarantee the normal speed of people’s flow passing through a millimeter-wave security instrument [19,20]. This real-time detection of a small-sized target makes the task more challenging. For real-time detection of concealed targets under people’s clothing, recently developed deep learning algorithms may be a better alternative. Concerning real-time detection requirements, undoubtedly a kind of one-step structure network is the best choice. Early work on the development of a one-stage object detector includes the OverFeat method [21]. Several object detection frameworks, such as Single Shot MultiBox Detector (SSD) and You Only Look Once (YOLO), which utilize anchors or grids to propose candidate object localization, are typical one-stage detection methods [22,23,24,25,26]. Recently, machine learning algorithms have been gradually applied to PMMW imagery and obtained the best results are by Random Forest (RF) algorithm with Haar features extracted from the preprocessed images [18]. In the meanwhile, neural network algorithms have been used in order to extract concealed targets for greater speed and accuracy. In Reference [2], research utilizing a context embedding object detection network showed effective results from AMMW images with massive sample data, which has been proved to be beneficial and gained 2% and 1% improvements on area under curve (AUC) and true positive (TP), respectively, compared with the PMMW data set using the same method. With the aid of deep learning, outstanding deep neural networks (DNNs) models are proposed so as to generate high precision, approximately 85%, human body profiles in PMMW images [6]. Additionally, YOLOv3 algorithms have been successfully applied for real-time detection of targets such as cars, rail surface defects, and airplanes and other things [27,28,29,30].

However, due to the performance of PMMW equipment, the deployment requirements of real-time algorithm, the detection accuracy and the complexity of the types of concealed objects on human body that need to be detected, the current detection methods often need to carry out with a large amount of data for data collection, and other more identification works [6,18]. Different from the existing research, in this paper, based on a small sample dataset (no more than 2000 images) and the characteristics of PMMW images, the YOLOv3 model were chosen to be applied in the real-time identification of identifying concealed weapons on the human body when the person moves through an indoor PMMW imaging system. The performance and accuracy of the real-time target detection capabilities were compared with the SSD algorithm with the same insufficient sample data. Aiming at practical application, the influence of clothing thickness, the detection accuracy, and the speed for concealed object detection were considered with the processing of PMMW images. Finally, through the test data acquired from the PMMW imaging system of Beihang University, China, it was verified the efficiency and positioning accuracy of real-time recognition for concealed weapons on the human body based on the YOLOv3 algorithm. The contribution of this paper lies in that using limited sample data collection and YOLOv3 algorithm to realize the satisfied real-time detection accuracy and effect for the application of recognizing small-size metal contraband from PMMW images.

This paper is organized as follows. In Section 2, the methodology of the detection of concealed weapon on the human body based on the YOLOv3 is presented. In Section 3, the experimental results and analyze based on the dataset of SAIR-U are presented with YOLOv3-13 and YOLOv3-53, respectively [19]. The last part, Section 4, is the conclusion.

## 2. Method

The YOLO target detection network is an end-to-end, one-step structure which was developed in recent years [23]. By learning from a large number of labeled images, the detected target bounding box (BB) and the categorical probability prediction can be directly obtained. It has a relatively fast and high mean average precision(mAP) performance to scale variations, since it adapts multiple convolution layers for multi-scale object detection [23]. As a real-time target detection algorithm, YOLOv3, involving a series of ideas of YOLOv1 and YOLOv2, has been optimized from a series of aspects from sample data, network structure design, and model training [24]. It uses the darknet-53 and darknet-13 networks for feature extraction. Similar to the Visual Geometry Group (VGG) network, it is mainly composed of a set of 3×3 and 1×1 convolutional layers. The YOLOv3 deepens the convolutional network to extract a greater number of deep features. A short-cut is added to YOLOv3 to build a residual module, thus avoiding gradient disappearance from deep network training. Secondly, it is introduced into the Faster R-CNN [31,32], with the idea of inputting the a priori anchor box during model training, but the selection of the anchor box is automatically obtained from the training data by means of k-means clustering [24].

**Figure 1:**
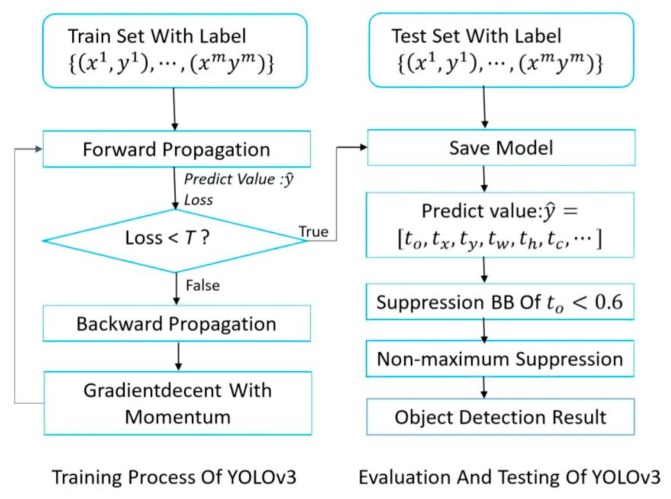
The flow chart of weapon detecting based on YOLOv3.

Aiming at real-time detection of a concealed weapon on the human body with YOLOv3, it is necessary to choose a special loss function and design the corresponding network structure. Through forward and backward propagation with gradient descent, training model parameters can be derived from inputted labeled sample data via continuous iteration [33]. The data processing flow is presented in Figure 1:

### 2.1. YOLOv3 Network Architecture

**Figure 2:**
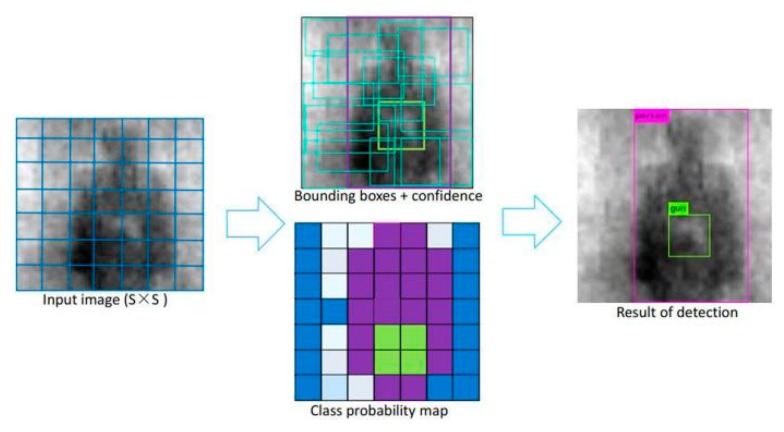
The YOLOv3 object detection method.

Redmon et al. originally proposed the YOLO target detection algorithm in 2016, based on many research works [23,33,34]. Different from the Regions with CNN features (R-CNN) series target detection algorithms, it treats the target detection task as a regression problem, and directly obtains the target bounding box, the confidence $P_{c}$, and the probabilities of being a certain target by taking all pixel values of the image as the input. As shown in Figure 2, it uniformly samples the input image of a size of 416 × 416, and supposes the image is segmented by 3 × 3 grids. Each grid predicts B bounding boxes, involving seven predicted values ($b_{x}$, $b_{y}$, $b_{w}$, $b_{h}$, and its corresponding $P_{c}$ and class probability, $c_{1}\;{\mathrm{and}\;c}_{2}$), and all predicted values are output as a tensor with a shape of 3 × 3 × (B × 7). Where $P_{c}$ indicates the confidence, defined as the target in the predicted bounding box; ($b_{x}$,$b_{y}$) indicates the position of the center point of the target relative to its corresponding grid; ($b_{w}$,$b_{h}$) represents the width and the length of the target, which are relative to the entire image; and ($c_{1}$, $c_{2}$) represents the category probabilities of two different targets. 

**Figure 3:**
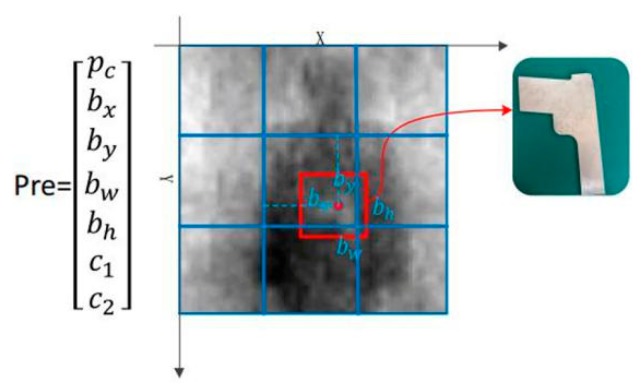
Bounding boxes prediction.

This method divides one image into an S × S grid. For each grid cell, B bounding boxes with confidence are predicted. These predictions are encoded as an S × S × (B × 7) tensor. As shown in Figure 3, PMMW image contraband target detection by YOLOv3 requires the detection of metal gun and human body targets. The model was able to detect three different scale targets for grid sizes of 13 × 13, 26 × 26, and 52 × 52, respectively, and can predict simultaneously three bounding boxes of each scale. When providing a PMMW image in the test, each grid outputs a 21 dimensional vector prediction value as follows:

(1) $$y_{\left(1,1\right)}^{1}={\left[p_{c_{1}}^{\left[1\right]},b_{x_{1}}^{\left[1\right]},b_{y_{1}}^{\left[1\right]},b_{w_{1}}^{\left[1\right]},b_{h_{1}}^{\left[1\right]},c_{1}^{\left[1\right]},c_{2}^{\left[1\right]},p_{c_{1}}^{\left[2\right]},\cdots ,c_{1}^{\left[3\right]},c_{2}^{\left[3\right]}\right]}^{T}$$

Therefore, the size of the predicted tensor is S × S × (B × (1 + 4 + 2)). The red border in Figure 3 represents the predicted bounding box of the metal contraband target, which is preserved by confidence threshold filtering and non-maximum suppression.

The design of the YOLOv3 target detection network structure used some inspiration from GoogLeNet [25]. The network structure was mainly built using 3 × 3 and 1 × 1 convolutional layers, and a residual layer was added to improve the learning ability of the network deepening network, as shown in Table 1 [35]. In this experiment, the input for the YOLOv3 network was a 416 × 416 × 3 tensor, and it can output three sensors in scales 13 × 13 × 21, 26 × 26 × 21, and 52 × 52 × 21, respectively, by adding a passthrough layer and an upsampling layer to the target detection process. 

The parameters of a convolution layer are set according to the YOLOv3 model. But parameters of detection layer are designed for detecting two objects (human body and weapon), according to YOLOv3‘s encoding of each input picture as shown in Table 1. And there are three grid sizes of 13, 26, and 52 for each picture. So, the parameters selections of those layers indicate to use these three grid scales of each picture, and each grid contains 21 predictions (include bounding boxes and category probability of two objects) defined as in equation (1). In this experiment, the input for the YOLOv3 network is a 416 × 416 × 3 tensor, and it can output three sensors in scales 13 × 13 × 21, 26 × 26 × 21, and 52 × 52 × 21 respectively, by adding a passthrough layer and an upsampling layer to the target detection process. 

### 2.2. Sample Data Augmentation

Vision-based deep learning model training should have high precision and strong generalize ability without a large number of training samples. The correlation, difference, and quantity of training samples can directly influence the efficiency of the training model. In most cases, the actual available samples are limited. In this case, some data expansion methods are often needed to cover this problem. The data expansion methods adopted in this paper include saturation adjustment, contrast adjustment, brightness adjustment, hue adjustment, etc. 

### 2.3. Anchor Boxes Prediction

**Figure 4:**
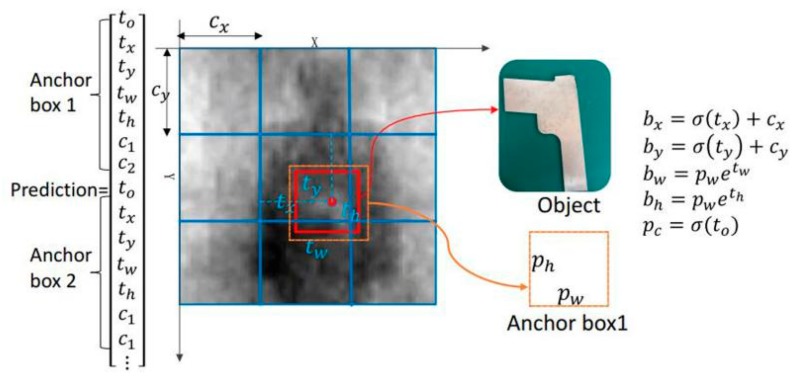
Prediction based on anchor boxes.

The YOLOv3 regards the anchor box as the a priori bounding box and imposes constraints on the predicted bounding box. The anchor Box optimizes the positioning accuracy of the bounding box. The prior anchor boxes are usually obtained by k-means clustering [24,26]. In this experiment, we assume that three prior anchor boxes are obtained through k-means, and the mesh size of the two-class network is 3 × 3. When predicting an input sample, each grid will predict three bounding boxes, with each bounding box containing five predicted values, which are the four coordinates $t_{x}$, $t_{y}$, $t_{w}$, $t_{h}$ and its corresponding $t_{o}$. At the same time, two class probability prediction values, $c_{1}$ and $c_{2}$, can also be predicted. That is to say, the overall output predicted values are 3 × 3 × 3 (1 + 4 + 2) three-dimensional tensor. As shown in Figure 4, assuming that the metal target prediction bounding box (red solid line) has the highest overlap with the a priori bounding box, anchor box1 (orange dotted line), and the grid of the bounding box is offset from the upper left corner of the entire image by $\left(c_{x},c_{y}\right)$, the width and length of anchor box1 are $p_{w}$ and $p_{h}$, respectively. Then, the actual bounding box positioning prediction value for the corresponding metal target is determined as [26]:

(2) $$\{\begin{array}{l} b_{x}=\sigma \left(t_{x}\right)+c_{x}\\ b_{y}=\sigma \left(t_{y}\right)+c_{y}\\ b_{w}=p_{w}e^{t_{w}}\\ b_{h}=p_{h}e^{t_{h}}\\ p_{c}=\sigma \left(t_{0}\right) \end{array}$$

Here, σ represents the sigmoid function. A kind of sigmoid constraint allows the prediction and positioning of bounding boxes to be robust.

### 2.4. Model Training and Evaluation

The most prominent feature distinguishing YOLOv3 from other methods is the realization of end-to-end detection [26,33]. The two target detection models of YOLOv3-13 and YOLOv3-53, with 13 and 53 layers, respectively, were trained in this experiment. During the model training, one of the hyperparameters, batch_size, was set to be 8, the momentum decrease parameter was β = 0.9, the learning rate value was set as 0.001, the learning rate attenuation decay was 0.0005, and the iterative training was executed on a computer with GPU-1080. Because of the YOLOv3-53 model, Darknet-53 achieved the highest measured floating-point operations per second. 

**Figure 5:**
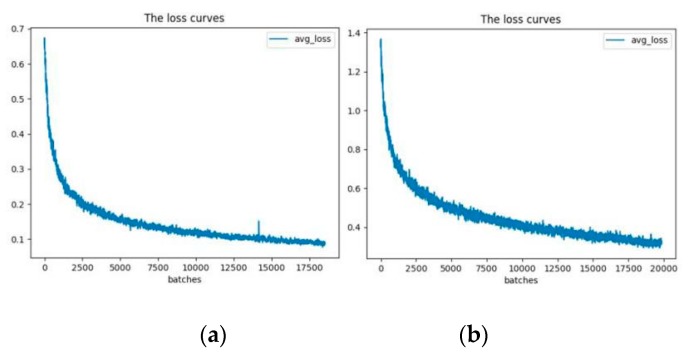
The curves of loss function with YOLOv3: (**a**) loss function for YOLOv3-53; (**b**) loss function for YOLOv3-13.

Utilizing the GPU can enable model learning speed, faster convergence, and time savings [24]. YOLOv3-13 and YOLOv3-53 were trained for nearly 8 h and 24 h, respectively, and their loss function curves are shown as in Figure 5. As shown in the above illustration, both of the loss values of the models decrease with the output batch data, and the loss values of the first 200 training batches have rapid decrements. When the model parameters are iteratively updated, the loss function of YOLOv3-53 tends to be gentle at 17,000 training batches, while that of YOLOv3-13 at 20,000 training batches. In addition, compared with that of YOLOv3-53, the loss function value of YOLOv3-13 has more and bigger fluctuations.

**Figure 6:**
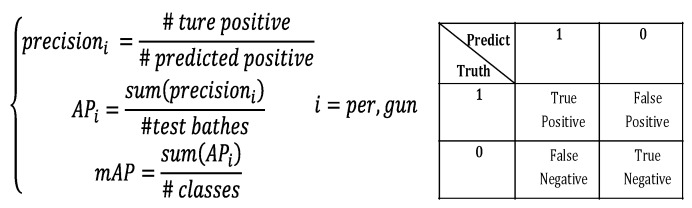
Calculation process of mAP.

As performance evaluation indicators, Intersection Over Union (IoU) and mAP are commonly used in target detection [36]. The IoU is often used for edge frame overlap or positioning accuracy evaluation. The higher the IOU value, the greater the overlap of the two bounding boxes and, correspondingly, the higher the positioning accuracy. On the other hand, mAP is an accuracy indicator that evaluates the accuracy of the target detection algorithm through setting the IoU threshold [24]. In the test phase, when the value of IoU satisfies IoU > 0.5, calculated from the predicted bounding box and the reference bounding box, the prediction of the bounding box will be an effective one. Then, we can calculate the mean accuracy of the target detection from all of the effective ones. During the experiment, mAP was taken as one important indicator to evaluate the effectiveness and performance of the trained YOLOv3 model. Firstly, 20% of the sample data was taken as a test set, and the selected batch size was 8 during the test. Furthermore, the prediction accuracy of the human body and the metal weapon was calculated for each batch. After all of the batches were complete, the average precision (AP) of the two categories was calculated separately. Finally, the mean progress (mean AP, mAP) of the human body and metal weapon detection was calculated. The actual calculation process is shown in Figure 6.

As shown in Figure 6, #true positive represents the sample quantity of the inputted positive sample batch of true values; #predicted positive is the sample quantity of the predicted positive sample batch; #test batches is the number of batches for testing; and #classes is the number of categories. During the experiment, through accuracy evaluation for two-category target detection, the mean accuracy ${\mathrm{AP}}_{i}$ was calculated first of all, and then the mAP value was obtained.

## 3. Experimental Result and Analysis

### 3.1. Experimental Dataset

The experimental data were a set of images obtained by a PMMW real-time imager from Beihang University, China [19,20], which are available as the Supplementary File, with a human carrying a metal gun in each image. In order to ensure the detection accuracy and generalization ability of the model, the sample data should be diversified. A total number of 1634 PMMW images were captured from the imager at the 34 Ghz band. To test the robustness of our approach, the sample data were collected by the examinee wearing different thicknesses of clothing, carrying the metal gun contraband at different temperatures and imaging speeds. This covered the effects of temperature, imaging speed, and clothing thickness on the detection target, in addition to the frequency band. The specific sample collection parameters are listed in Table 2.

**Figure 7:**
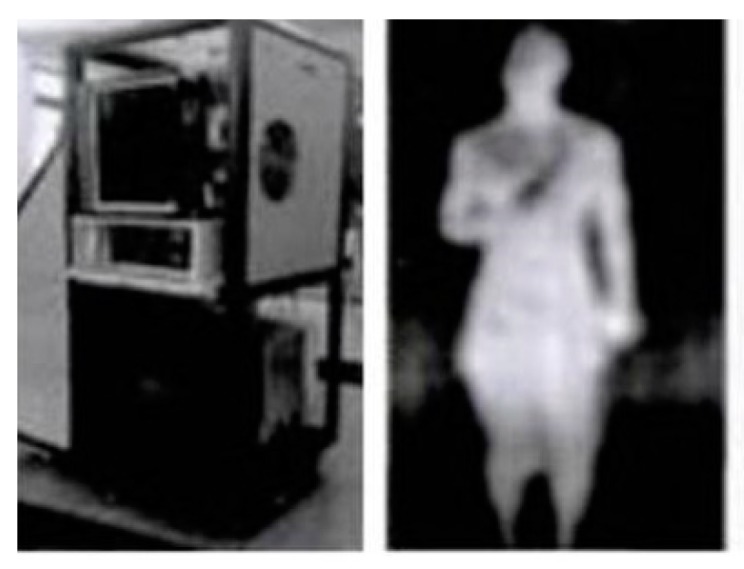
The SAIR-U system and detected image.

The PMMW real-time imager, SAIR-U, was developed by the Microwave Laboratory of Beihang University (see Figure 7). It has been used in non-contact, non-cooperative (i.e., no need for a fixed posture) security, especially in environments of large passenger flow. The basic design indicators of this PMMW system involve the following aspects:

- The working band is the Ka band, at 34 Ghz;
- The imaging field of view is designed to be 40 × 22°;
- The imaging distance range is set to 2.5~5 m;
- The sensitivity to temperature is 1~ 2 K.

During the data collection procedure, the tester wore clothes of different thicknesses and carried contraband which was placed inside of the garment, and passed through the PMMW imaging system at a certain speed. At the same time, considering the impact of the human body’s movement on data quality, the PMMW image data at a fixed imaging distance (3 m) were also collected. Because the scene temperature, the thickness of the clothing, and the imaging distance were different, the imaging speed had a certain influence on the image quality. When collecting the experimental data, the temperature was set to 10 °C, 24 °C, and 37 °C, respectively; the imaging speed was set to 10 frames per second (FPS), 15 FPS, and 25 FPS, respectively; and the imaging distance was about 2.5~5.0 m. 

**Figure 8:**
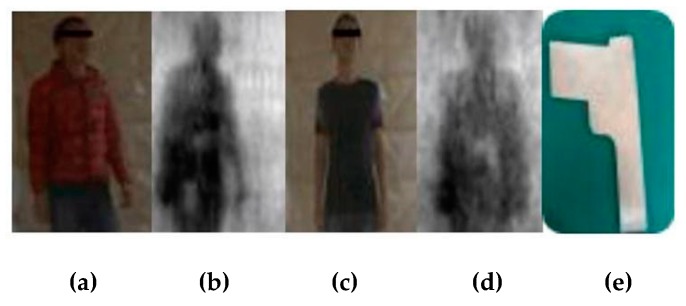
Data source and sample of contraband: (**a**) optical image with thick clothes; (**b**) PMMW image; (**c**) optical image with thin clothes; (**d**) PMMW image with concealed gun; (**e**) sample of metal gun.

Figure 8a,c shows the optical images of the data collected by the examinee wearing different thicknesses of clothing. Figure 8b,d represents the corresponding PMMW images. According to the reflection characteristics of the materials in the ka-band, the human body’s reflectivity is lower than that of the metal, so the white block with high reflectivity on the human body in the image is the contraband of the experimental gun carried by the examinee.

**Figure 9:**
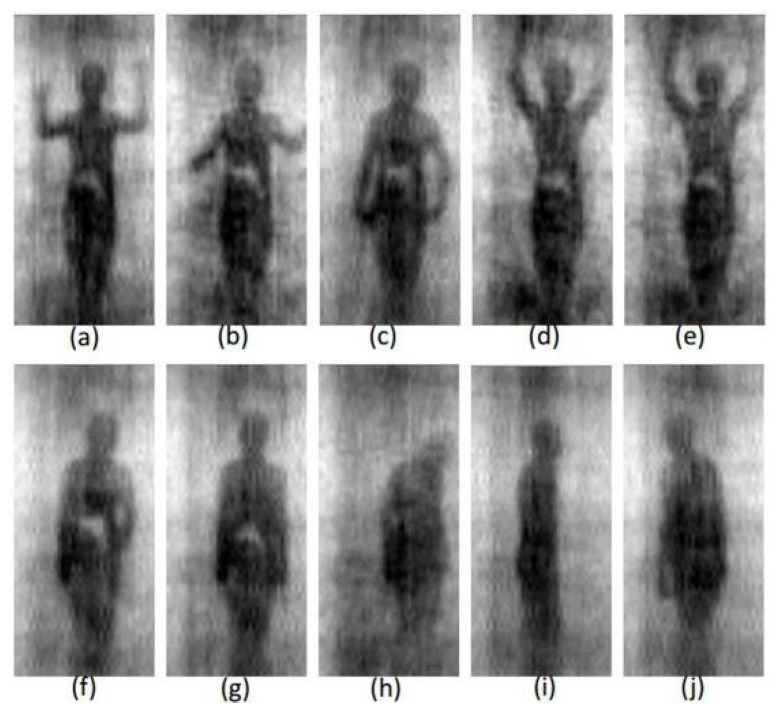
Different samples of experimental data (**a**−**j** represent the different walking states as the tester passing through the SAIR-U system).

The sample dataset mainly comprised the visual contraband samples (Figure 9a–g), invisible contraband samples (Figure 9h–j, in which the contraband information could not be obtained due to the rotation of the human’s body), and a small number of noise samples. During the experiment, the labeled 1634 sample data were divided into training and test sets according to the ratio of 8:2. The labeled training set was used to iteratively update the model parameters, and the tag test set was used for model accuracy evaluation after model training.

### 3.2. Evaluation and Result Analyses

To evaluate the performance of YOLOv3 for concealed weapon detection, several analyses were designed. 

#### 3.2.1. Setting of the Number of Convolution Layers

In this experiment, we tested images of contraband detection at different temperatures and different clothing thicknesses. Experimental results show that reducing the number of convolution layers of the YOLOv3 model results in fewer parameters, i.e., fewer features are extracted; therefore, this also reduces the detection accuracy of PMMW images. Thus, in practical applications, it is necessary to make a trade-off between detection accuracy, detection speed, and model parameter quantity according to practical requirements. 

The model parameters of YOLOv3-13 and YOLOv3-15 are trained in the computer system as Ubuntu16.04, Intel I7 9700K, 32GB of memory, GeForce GTX 1080Ti with 12GB memory, from scratch. The loss functions all use multiple loss functions, that is, confidence loss and positioning loss. Specific hyperparameters, the former is iterated 20,000 times for all train sets with batch_size 8, momentum: 0.9, weight_decay: 0.0005, base_lr: 0.001, training time is nearly 24 hours, and the latter is iterated 17,000 times for all train sets with batch size 8, momentum: 0.9, weight_decay: 0.0005, base_lr: 0.001, training time is nearly 8 hours.

**Figure 10:**
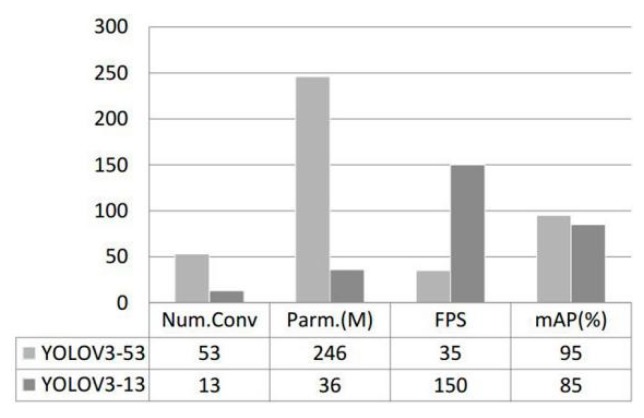
The experiment results, in terms of speed and precision, of different networks, including YOLOv3-53 and YOLOv3-13.

The number of convolution layers, parameter quantities, detection speeds, and mAPs of YOLOv3-13 and YOLOv3-53 after model training are shown in Figure 10. As the results show, the YOLOv3-13 model uses a 13-layer convolution network with an 85% mAP on the individual test set. However, it can reach the highest 150 FPS detection speed and a minimum number of 36M parameter quantities. By contrast, the YOLOv3-53 model uses a 53-layer convolution network, which has higher mean detection accuracy than the former, but its detection speed is only 35 FPS, and the parameter amount is as high as 246 M.

**Figure 11:**
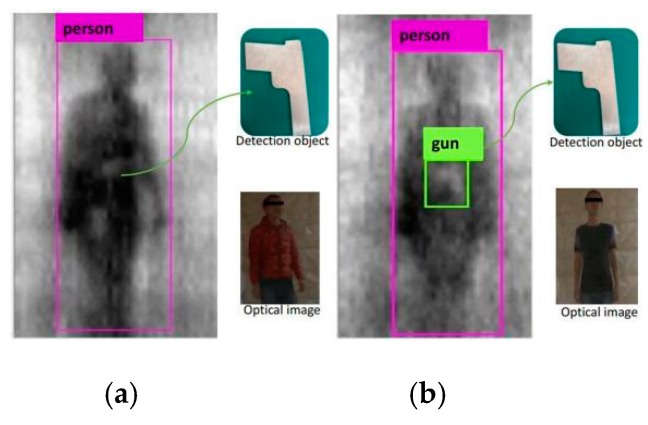
Detection of two-category test images with YOLOv3-53: (**a**) The test result from the tester wearing thick cloths; (**b**) the test result from the tester wearing thin cloths.

**Figure 12:**
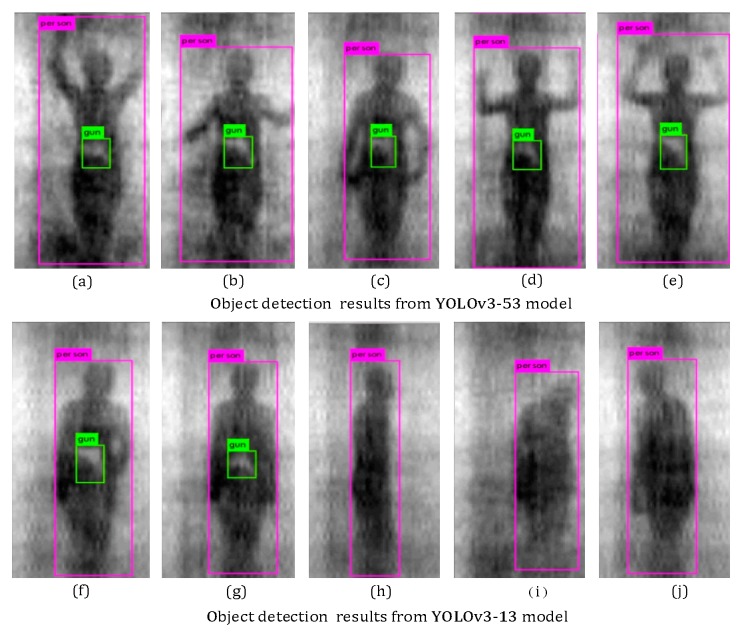
Detection results of a gun from human body with YOLOv3-53 (**a**–**e**) and YOLOv3-13 (**f**–**j**).

Figure 11 shows the qualitative results of the target detection, with the tester wearing different thicknesses of clothing, from the PMMW images of the YOLOv3-53 model. The test results show that the YOLOv3-53 model can stably detect metal contraband on the human body in PMMW images, when the tester is wearing thinner clothing, and it normally has a real-time detection speed as shown in Figure 11b. However, due to the human body’s moving and rotation and the interference from the thick clothing on the millimeter wave radiation, the metal contraband in the PMMW image has fewer features, and the YOLOv3-53 model is almost unable to detect the metal contraband (as shown in Figure 11a. Furthermore, the complete experimental results regarding the human body and metal weapon detection is shown in Figure 12, including that from a series of body postures when a person moves normally through the indoor PMMW imager.

The experimental results show that both the YOLOv3-13 and YOLOv3-53 models have real-time target detection capabilities, as shown in Figure 12. Among the results, YOLOv3-53 has more advantages with higher precision from the aspect of accuracy requirements. However, according to the later requirements of model deployment, YOLOv3-13, with a lower parameter quantity, can reduce the calculation pressure.

The experiment results of the real-time detection of concealed guns on human body are shown as Figure 12. 

#### 3.2.2. Robustness in Relation to Room Temperature

To test the effect of different indoor temperatures, this experiment chose passive millimeter waves, carrying the contraband in the body of 10–40 °C as test data. The test results of the target detection of millimeter wave contraband at different temperatures are shown in Figure 12. The results show that the indoor temperature of 10–40 °C had little effect on the radiation of passive millimeter waves and did not reduce the detection accuracy of contraband.

#### 3.2.3. Robustness in Relation to the Clothing Thickness

To test the effect of the thickness of clothing, the experiment used clothing thicknesses of 3~5 mm and 10 mm or more to cover the contraband for the passive millimeter wave test. Figure 11a,b shows the contraband detection results of clothing thicknesses of more than 10 mm and less than 10 mm, respectively. The results showed that the thickness of clothing above 10 mm weakens the radiation of the millimeter wave, which leads to the occurrence of contraband inspection.

#### 3.2.4. Efficiency Analyses 

**Figure 13:**
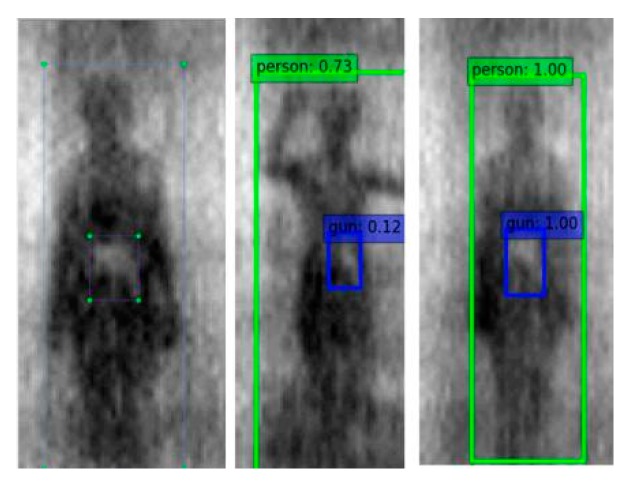
Detection results of a gun from human body with SSD-VGG16 algorithm.

To verify the efficiency of YOLOv3 in real-time detection for PMMW images with a small sample dataset, this paper conducted another experiment with the same sample dataset and the SSD-VGG16 algorithm, a kind of one-step structure deep learning algorithm like YOLOv3. We adopt the SSD algorithm of Caffe version of no additional data expansion. All parameters were initially default parameters of SSD algorithm. The training times were 120,000 times, gamma: 0.1, momentum: 0.9, weight_deck: 0.0005, base_lr: 0.0001, and the training time of 12W times was about 12 hours. Machine configuration: Intel i7 9700K, memory 64 GB, graphics card RTX 2080ti, 11GB, training system Ubuntu 16.04. Part of the experiment results executed with SSD algorithm have been shown in Figure 13.

The final results show that YOLOv3-13 and SSD-VGG16 had a nearby detection mean average accuracy of metal guns in the PMMW images, but the former had a higher detect speed with 150FPS than later with 28FPS. In addition, YOLOv3-53 had the largest detection mean average accuracy among them. From the above comparative experimental results, it can be verified that YOLOv3-53 had a better identifying accuracy and efficiency than YOLOv3-13 and SSD-VGG16 (see Table 3).

In this experiment, SSD used VGG-16 as the basic network of feature extraction, while YOLOv3-53 used darknet-53 as the basic network of feature extraction. Although VGG-16 and darknet-53 mainly use convolutional layers to extract the features of the picture, the design of the residual block is used in the latter to make it learn deeper feature information than the former. In addition, the input resolution of the SSD-VGG16 algorithm is 631x284, and each image predicts 24,564 bounding boxes when detecting. While the input resolution of the YOLOv3 algorithm is 416 × 416, the images predict 3549, 14,196, and 56,784 bounding boxes with three scale 13× 13, 26× 26 and 52× 52 during detecting processing.

**Figure 14:**
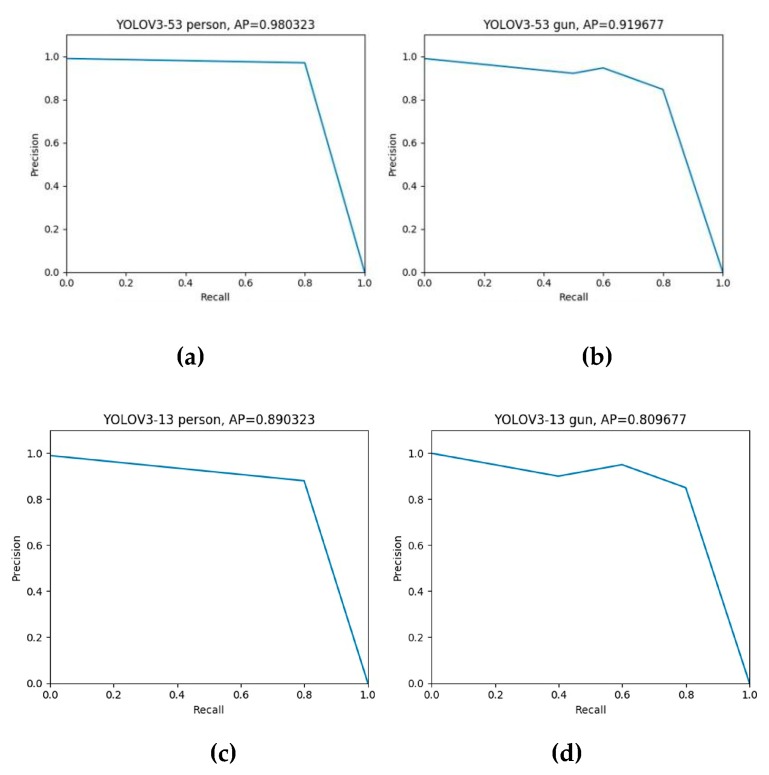
The precision-recall curves of YOLOv3-53 (**a**−**b**) and YOLOv3-13 (**c**−**d**) for detecting person and gun respectively.

**Figure 15:**
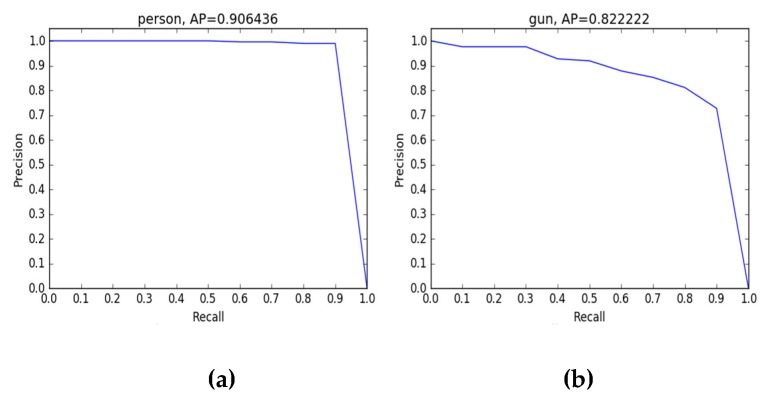
The precision-recall curves of SSD-VGG16 for detecting person (**a**) and gun respectively (**b**).

The precision-recall curves of YOLOv3-53, YOLOv3-13, and SSD-VGG16 are shown in the Figure 14 and Figure 15. For the human body and the gun, the average accuracy is 98%, 91%, 89%, 80%, 90%, and 82%. The results show that compared with the other two, the YOLOv3-53 algorithm can maintain detection accuracy of 98% and 91% for humans and prohibited guns with the increase of the recall rate. It was verified that the YOLOv3-53 algorithm had a better performance in identifying contraband than the other two.

Meanwhile, the experiment results from the above also show that the following conclusions, which have certain reference significance of the real-time detection of contraband on human body for PMMW images:

Computational complexity: The floating-point operations per second (FLOPS) for YOLOv3-53, YOLOv3-13, and SSD-VGG16 were about 37.7 billion, 15.5 billion, and 21.6 billion, respectively. The parameters of YOLOv3-53, YOLO3-13, and SSD-VGG16 are 246M, 36M, and 92M, respectively. From the perspective of computing resource consumption, YOLOv3-13 was better compatible with small computing devices if we only consider the computing resources.

Considering the purpose of the paper and application requirements for PMMW systems, Both the YOLO and SSD algorithms can reach a processing speed of more than 24 frames per second, which can meet the real-time application requirements for the imaging capabilities of existing equipment. But considering the object detection requirements for practical scenarios, the parameters of YOLOV3-53 are a better choice than the others.

Even when the sample data of PMMW images are not sufficient, comparing with the existing similar algorithms for PMMW images [6,18], both the YOLO and SSD algorithms still maintain a high detection accuracy of concealed metal contraband on the human body, especially small contraband hidden in the body. This has certain significance for the practical collection of sample data and the training of PMMW equipment.

## 4. Conclusions

Different from traditional metal security gates and X-ray detectors, non-contact and non-cooperative PMMW imagers have become a primary choice for security checks in large public places. In the meantime, the YOLO algorithm is also an excellent real-time detection method of great development potential [37,38,39,40]. This paper focused on the real-time metal contraband detection from human body for PMMW images with a small sample dataset and YOLOv3 algorithms. The Yolov3-13 and Yolov3-53 target detection models with different convolutional layers were trained, and their advantages and disadvantages were analyzed, and the experiment results were compared with that of SSD algorithms. The experiments shown that the YOLOv3-based contraband detection method for the PMMW images and can meet the real-time detection requirements during large passenger flows. In terms of trade of detection accuracy, detection speed, and computation resource, the YOLOv3-53 model is more advantageous and effective, even with an insufficient sample data. Due to the equipment limitations, the available multi-source PMMW image data are limited, and the data needs to be detected and improved. Furthermore, more training tests of various types of contraband should be developed for further PMMW security requirements.

## Figures and Tables

**Table 1:** The core network architecture for the target detection of YOLOv3. (the table omits upsampling layer and passthrough layer).

Type	Filters	Size/Stride	Output	
Convolutional	32	3 × 3/1	416 × 416 × 32	
Convolutional	64	3 × 3/2	208 × 208 × 64	
Convolutional	32	1 × 1/1	208 × 208 × 32	
Convolutional	64	3 × 3/2	208 × 208 × 32	×1
Residual			208 × 208 × 64	
Convolutional	128	3 × 3/2	104 × 104 × 128	
Convolutional	64	1 × 1/1	104 × 104 × 64	
Convolutional	128	3 × 3/1	104 × 104 × 128	×2
Residual			104 × 104 × 128	
Convolutional	256	3 × 3/2	52 × 52 × 256	
Convolutional	128	1 × 1/1	52 × 52 × 128	
Convolutional	256	3 × 3/1	52 × 52 × 256	×8
Residual			52 × 52 × 256	
Convolutional	512	3 × 3/2	26 × 26 × 512	
Convolutional	256	1 × 1/1	26 × 26 × 256	
Convolutional	512	3 × 3/1	26 × 26 × 512	×8
Residual			26 × 26 × 512	
Convolutional	1024	3 × 3/2	13 × 13 × 1024	
Convolutional	512	1 × 1/1	13 × 13 × 512	
Convolutional	1024	3 × 3/1	13 × 13 × 1024	×4
Residual				
Convolutional	512/256/128	1 × 1/1	13/26/52 × (13/26/52) × 512/512/128	
Convolutional	1024/512/128	3 × 3/1	13/26/52 × (13/26/52) × 1024/512/128	
Convolutional	21	1 × 1/1	13/26/52 × (13/26/52) × 21/21/21	
Detection			13/26/52 × (13/26/52) × 21/21/21	

**Table 2:** Data source of the PMMW imager, SAIR-U.

Temperature (°C)	Imaging Speed (FPS)	Resolution	Imaging Range	Number of Sample Images
10	(10, 15, 25)	5–6 cm @ 3 m	2.5–5.0 m	525
24	(10, 15, 25)	5–6 cm @ 3 m	2.5–5.0 m	540
37	(10, 15, 25)	5–6 cm @ 3 m	5.5–5.0 m	545

**Table 3:** Performance comparison between YOLO and SSD algorithms with the same dataset.

Algorithm	Number of Convalutional Layers	Parameter of Weights	FPS	mAP(%)
YOLOv3-53	53	246	35	95
YOLOv3-13	13	36	150	85
SSD-VGG16	35	92	28	86

## Supplementary Materials

The following are available at https://www.mdpi.com/1424-8220/20/6/1678/s1: sensors-20-01678-PMMW- data.zip.
